# Vacuum compression-molded polyvinyl alcohol microneedles for sustained three-day transdermal delivery of palonosetron hydrochloride

**DOI:** 10.1007/s13346-025-01980-z

**Published:** 2025-09-23

**Authors:** Meheli Ghosh, Sharvari M. Kshirsagar, Thomas Kipping, Ajay K. Banga

**Affiliations:** 1 Center for Drug Delivery Research, Department of Pharmaceutical Sciences, College of Pharmacy, Mercer University, 3001 Mercer University Drive, Atlanta, GA 30341 USA; 2 MilliporeSigma a Business of Merck KGaA, Frankfurter Strasse 250, 64293 Darmstadt, Germany

**Keywords:** Microneedles, Transdermal, Palonosetron hydrochloride, Sustained drug delivery, Vacuum compression molding

## Abstract

This study introduces vacuum compression molding (VCM) as a novel, solvent-free method for fabricating palonosetron hydrochloride (PAL HCl)-loaded polyvinyl alcohol (PVA) microneedles (MNs), addressing limitations of conventional micromolding such as extended drying times, batch variability, and solvent residues. PAL HCl-a hydrophilic 5-HT3 receptor antagonist (MW: 332.85 g/mol) with a low therapeutic dose-was selected for its clinical relevance in managing chemotherapy-induced nausea and vomiting (CINV). The microneedle platform offers advantages over PAL HCl’s existing oral and injectable dosage forms, including pain-free application and improved patient compliance. The aim of this research is to develop and evaluate a scalable VCM-based fabrication approach for PAL HCl-loaded PVA microneedles, with the goal of achieving sustained, three-day in vitro transdermal drug delivery for improved CINV management. Ten PVA grades (varying in molecular weight and viscosity) were screened to optimize microneedle fabrication. Three formulations-M1 (particle-engineered PVA 4–88), M4 (PVA 5–88), and M5 (PVA 8–88)-demonstrated optimal mechanical strength, uniform geometry (SEM imaging), and reliable skin penetration (~ 200 μm depth in dermatomed human skin). Physicochemical characterization (FTIR, DSC) confirmed the amorphous state of PAL HCl within the PVA matrix and the absence of chemical interactions. In vitro release testing revealed biphasic profiles: an initial burst release for 8 h followed by sustained release over 72 h. Cumulative release inversely correlated with PVA molecular weight and viscosity, with M1 achieving 100% release, compared to M4 (74%) and M5 (67%). Permeation studies demonstrated M1’s superior performance (257.56 ± 29.73 µg/sq cm), exceeding passive diffusion by 8.8-fold and significantly outperforming M4 (64.99 ± 30.23 µg/ sq cm) and M5 (39.03 ± 20.20 µg/sq cm). These results validate VCM as a scalable, tunable platform for fabricating PAL HCl-drug-loaded microneedles, offering sustained transdermal delivery with clinical potential for CINV management.

## Graphical Abstract

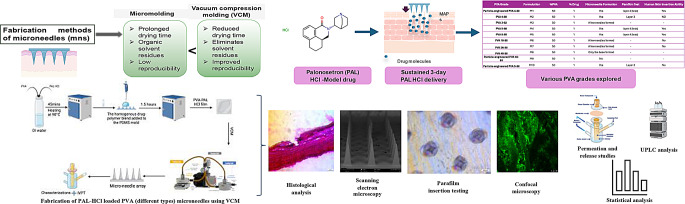

## Introduction

 Transdermal drug delivery enables systemic administration of therapeutics through the skin, bypassing gastrointestinal degradation and hepatic first-pass metabolism while offering controlled release and improved patient adherence [1]. However, the stratum corneum, the outermost skin layer composed of keratin-rich corneocytes and lipid bilayers-acts as a formidable barrier, restricting majority of therapeutically active compounds from passively permeating into the skin’s layers [1, 2]. Hydrophilic drugs, biologics, and macromolecules face significant permeation challenges due to the stratum corneum’s hydrophobic nature, which limits their ability to partition into the lipid-rich skin matrix [3].

To overcome these barriers, chemical and physical enhancement strategies have been developed. Chemical permeation enhancers modify skin structure by disrupting lipid organization or increasing fluidity, while physical methods like microneedles (MNs), iontophoresis, and electroporation create transient aqueous pathways [4, 5]. Among these, MNs have emerged as a minimally invasive solution for delivering hydrophilic small molecules, peptides, and vaccines [6].

Microneedles have micron-scale needle arrays designed to penetrate the stratum corneum, creating transient hydrophilic microchannels that enable efficient transdermal delivery of hydrophilic molecules, peptides, proteins, and vaccines [7]. By targeting the epidermis without stimulating deeper dermal nerve endings, MNs provide a minimally invasive and pain-free alternative to hypodermic injections [8, 9]. MNs are categorized into solid, hollow, coated, dissolving, and hydrogel-forming types, each tailored for specific applications. While MNs can be fabricated from metals, silicon, or polymers, polymeric variants have gained prominence due to their biodegradability, biocompatibility, and tunable drug release kinetics [6, 8, 10].

Conventional polymeric microneedle production relies on solvent-based micromolding, where polydimethylsiloxane (PDMS) molds are filled with polymer solutions (e.g., PVA, poly(lactic-co-glycolic) acid (PLGA), etc.) and subjected to centrifugation or vacuum-assisted settling, followed by extended drying cycles (3–5 days) [11, 12]. However, this method introduces challenges such as residual solvents, structural defects, extended drying time, and scalability limitations due to multi-step processes and inconsistent reproducibility [9, 13].

Vacuum compression molding (VCM) has emerged as a solvent-free alternative to address these issues. In this process, thermoplastic polymers are melted under vacuum, compressed into molds, and rapidly cooled to form mechanically robust microneedle arrays. For instance, MeltPrep^®^ VCM employs polytetrafluoroethylene (PTFE)-lined chambers with precise thermal control to produce microneedles featuring sharp tips and high aspect ratios. By eliminating solvents and reducing fabrication time to under two hours, VCM enhances scalability, making it a promising candidate for industrial applications. Despite these advancements, the application of VCM for drug-loaded microneedles remains unexplored [13].

Chemotherapy-induced nausea and vomiting (CINV) remains a debilitating side effect of cytotoxic chemotherapy, significantly impairing patient quality of life and threatening adherence to cancer treatment regimens. The pathophysiology of CINV involves activation of 5-HT₃ receptors in the gut and brainstem by serotonin released from enterochromaffin cells, triggering emetic reflexes. Palonosetron hydrochloride (PAL HCl), a second-generation 5-HT₃ antagonist having MW 332.86, log P 2.5, is highly potent and freely water soluble, making it a perfect model drug for transdermal MN delivery. PAL HCl has emerged as a first-line therapy due to its high receptor-binding affinity (>30-fold greater than first-generation agents) and allosteric binding mechanism, which collectively enable efficacy against CINV [12, 14].

Despite its therapeutic benefits, current PAL HCl administration routes, which are limited to intravenous injections and oral tablets, face critical limitations. Intravenous delivery is painful and requires clinical supervision, increasing healthcare burdens for patients already managing complex treatment schedules, while oral formulations suffer from erratic absorption due to chemotherapy-induced gastrointestinal dysfunction and first-pass metabolism [15–17]. These challenges are compounded by the extended emetogenic risk period of many chemotherapies, which often spans 72 h post-administration. A sustained-release transdermal system could provide continuous drug delivery over this critical window, bypassing gastrointestinal variability and enabling outpatient management [10, 18].

Polymeric dissolving MNs represent one of the most versatile transdermal delivery systems, with growing research interest over the past two decades due to their adaptability and patient-centric design [4]. Polyvinyl alcohol (PVA), a synthetic polymer, has emerged as a preferred material for these systems owing to its slow-dissolving properties, high water solubility, biodegradability, and biocompatibility [9, 19]. Listed as Generally Recognized as Safe (GRAS) by the U.S. FDA for pharmaceutical applications- PVA’s high molecular weight ensures minimal skin permeation, allowing natural elimination via epidermal turnover. Its water-soluble nature enables controlled matrix degradation upon contact with interstitial fluid, facilitating sustained drug release. PVA’s low immunogenicity and nontoxic profile make it ideal for prolonged skin contact. PVA-based dissolving MNs represent an ideal approach for this application, providing good mechanical strength to form robust microneedles that dissolve intradermally, releasing drugs directly into the microvasculature [19].

This study pioneers the application of VCM to fabricate PAL-HCl-loaded PVA MNs for sustained transdermal delivery. Ten PVA grades (PVA 4–88, Particle-engineered PVA 4–88, Particle-engineered PVA 3–82, PVA 5–88, PVA 8–88, PVA 18–88, PVA 26–88, PVA 40–88, Particle-engineered PVA 40–88, Particle-engineered PVA 5–88), differing in viscosity and molecular weight, were systematically investigated to assess their influence on MNs’ mechanical performance, structural uniformity, and skin penetration efficacy. A comprehensive evaluation of in vitro drug release and permeation profiles over a three-day period enabled direct comparison of the functionality and sustained-release capabilities across PVA grades. The results demonstrate the feasibility of slow-dissolving PVA microneedle systems to achieve prolonged transdermal delivery of PAL HCl, maintaining therapeutic plasma levels consistent with the 72-hour emetogenic risk window of cytotoxic chemotherapy. To our knowledge, this represents the first successful utilization of VCM for solvent-free fabrication of drug-loaded PVA microneedles capable of three-day drug release, offering a scalable alternative to invasive or frequent dosing regimens for CINV management.

## Materials and methods

### Materials

Palonosetron hydrochloride (PAL-HCl) was sourced from Ambeed (Arlington Heights, IL, USA). Pyramidal stainless-steel microneedle master templates (10 × 10 array; base dimensions: 150 μm × 150 μm, height: 500 μm) were acquired from Micropoint Technologies Pte Ltd (Singapore). Silicone elastomer components (Sylgard^®^ 184 base and curing agent) were procured from Dow Corning (Midland, MI, USA). Different PVA types/grades (PVA 4–88, Particle-engineered PVA 4–88, Particle-engineered PVA 3–82, PVA 5–88, PVA 8–88, PVA 18–88, PVA 26–88, PVA 40–88, Particle-engineered PVA 40–88, Particle-engineered PVA 5–88) were obtained as gift samples from Merck KGaA (Darmstadt, Germany). Dermatomed human cadaver skin was obtained from a tissue bank (New York, USA). HPLC-grade solvents were purchased from Pharmaco-AAPER (Brookfield, CT, USA). Methylene blue staining dye and Fluoresoft-0.35%^®^ were acquired from Sigma-Aldrich (St. Louis, MO, USA) and Holles Laboratories Inc. (Cohasset, MA, USA), respectively.

### Methods

#### Quantitative analysis

A UPLC method was developed for the quantitative determination of PAL HCl. Chromatographic separation was performed using a Waters Acquity UPLC HSS C18 column (100 mm × 2.1 mm, 1.7 μm) maintained at 35 °C, with a Waters Acquity sample manager FT-H and PDA detector (Milford, MA, USA). The mobile phase consisted of acetonitrile containing 0.1% trifluoroacetic acid and deionized water with 0.1% trifluoroacetic acid, mixed in a 35:65 (v/v) ratio, and delivered at a flow rate of 0.5 mL/min. The injection volume was set at 2 µL. PAL HCl was detected at 240 nm, with a retention time observed between 1.9 and 2.3 min.

Calibration standards were prepared in methanol using serial dilutions to cover a concentration range of 0.1 to 50 µg/mL. The method was validated for linearity, intraday and interday accuracy and precision, as well as for the limits of detection (LOD).

### Poly dimethyl siloxane (PDMS) mold fabrication protocol

Sylgard^®^186 silicone elastomer base and curing agent were combined at a 10:1 (w/w) ratio and manually mixed using a glass rod. Individual master structures were positioned in a 6-well plate and covered with the prepared PDMS mixture. To ensure bubble-free molds, the assembly was subjected to vacuum at 200 mbar (25 °C, 15 min) in a vacuum drying oven (Binder, GmbH, Germany). Subsequent thermal curing occurred at 90 °C for 3 h to achieve crosslinked PDMS molds. After cooling, the cured molds were manually demolded from the master structures and further punched to dimensions compatible with the MeltPrep^®^ VCM sample chamber to use for MN fabrication [13].

### Fabrication of PAL HCl-loaded PVA films

PVA films containing PAL HCl were prepared by dissolving various grades of PVA and PAL HCl in deionized water. The mixture was heated at 90 °C for 45 min in a hot air oven to ensure complete dissolution, followed by mixing on a rotary shaker to achieve a homogeneous solution. This drug-polymer blend was then cast into molds. The molds containing the casted drug-polymer blend were subjected to a second heating step at 90 °C for 1.5 h to facilitate water evaporation and film formation.

### Slide crystallization

To assess the physical stability of PAL-HCl in PVA blends, formulations containing 1%, 2%, 3%, 4%, and 5% (w/w) PAL-HCl in various PVA grades were prepared in deionized water. Each blend was cast onto glass microscope slides and dried overnight in a fume hood to allow solvent evaporation. The dried samples were examined each day for up to 3 days, under a Leica DM 750 optical microscope (Leica Microsystems Inc., Buffalo Grove, IL, USA) to detect any drug crystallization.

### Fabrication of PAL HCl-PVA microneedles using VCM

**Fig. 1:**
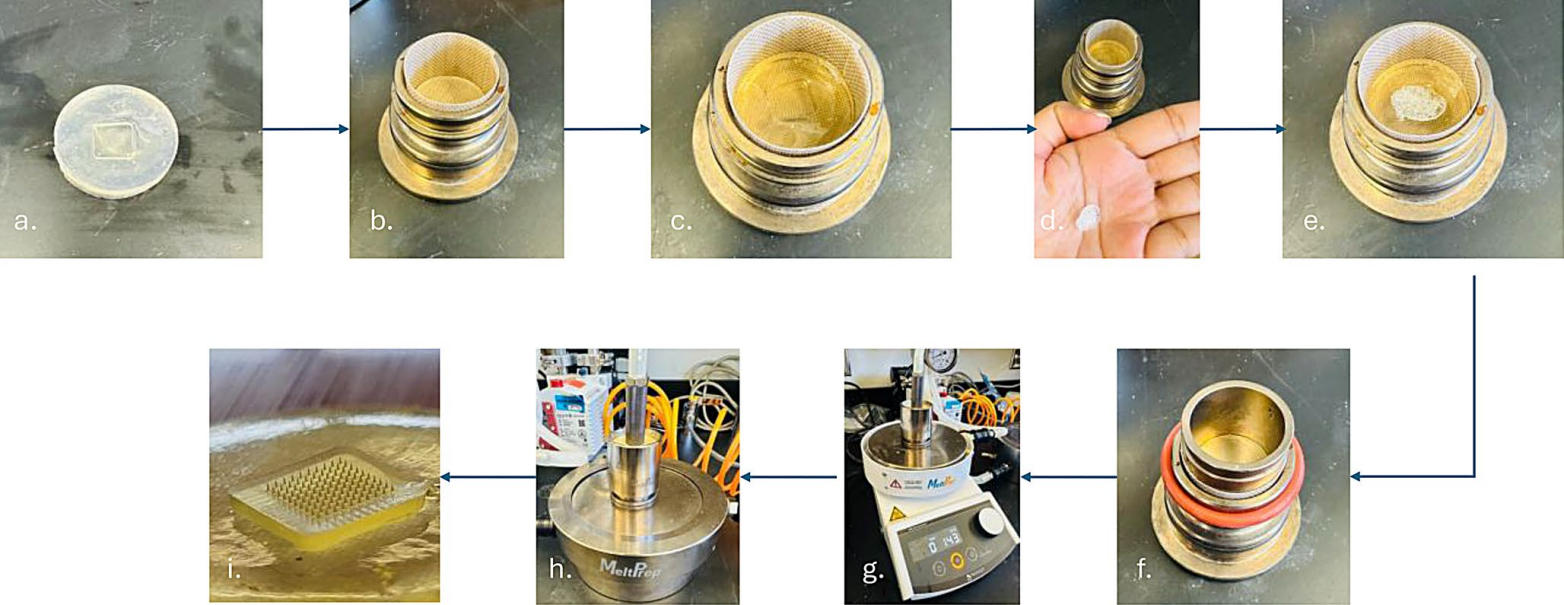
Step-by-step schematic of the Vacuum Compression Molding (VCM) process used for fabricating polymeric microneedles: (**a**) PDMS molds, (**b**) lining the sample chamber with PTFE foils, (**c**) placement of PDMS molds into the chamber, (**d**) PAL HCl-PVA film, (**e**) insertion of PAL HCl-PVA film into the VCM sample chamber, (**f**) positioning the piston over the sample wrapped in PTFE foils, (**g**) placing the sample chamber onto the heating platform under vacuum, (**h**) cooling phase, and (**i**) final PAL HCl-PVA microneedle array

PAL-HCl-loaded PVA microneedles were fabricated using the MeltPrep^®^ VCM system as depicted in Fig. 1. Ten PVA grades— PVA 4–88, Particle-engineered PVA 4–88, Particle-engineered PVA 3–82, PVA 5–88, PVA 8–88, PVA 18–88, PVA 26–88, PVA 40–88, Particle-engineered PVA 40–88, Particle-engineered PVA 5–88 (M1-M10, respectively)- were screened for microneedle fabrication feasibility using VCM. A pre-punched PDMS mold, prepared as described in previous sections, was inserted into the VCM tool’s molding chamber (Fig. 1c). A solvent-free PAL HCl-PVA film was positioned on the top of the PDMS mold, and polytetrafluoroethylene (PTFE) foil was applied to the chamber walls to prevent polymer adhesion during processing. The assembly was sealed under a vacuum of 15 psi (-103 kPa) and heated at 260 °C for 15 min on a temperature-controlled plate.

Following heating, the VCM tool was transferred to a cooling unit utilizing compressed air for rapid cooling to ambient temperature over 15 min. Continuous vacuum pressure ensured uniform thermal contact between the tool and heating/cooling surfaces, minimizing structural defects. After cooling, the drug-loaded MN array was manually demolded from the PDMS template and stored in desiccators at room temperature to maintain stability prior to further studies.

### Parafilm M^®^ insertion testing

To evaluate the insertion efficiency and mechanical consistency of the PAL-HCl-PVA MN arrays, a Parafilm M^®^ skin-mimicking model was used. The Parafilm was folded into ten layers to simulate skin resistance. The MN arrays were manually pressed onto the layered film with consistent force for 2 min and then carefully detached. The number of penetrated layers and insertion marks was analyzed using a Leica DM 750 optical microscope (Leica Microsystems Inc., Buffalo Grove, IL, USA) equipped with a 10x objective lens.

### Dye-binding studies for microchannel evaluation

Dye-binding analysis was conducted to assess the uniformity and integrity of microchannels created by PAL-HCl-PVA microneedles in dermatomed human skin. Following microneedle application, the treated skin was stained with a 1% (w/v) methylene blue aqueous solution for 2 min. Residual dye was blotted using Kimwipes, followed by gentle swabbing with 70% isopropyl alcohol to remove superficial stain while retaining dye within the microchannels. The stained skin samples were visualized, and digital images were obtained to determine effective skin microporation.

### Drug content of the microneedles

The PAL-HCl content in PVA MNs was determined via dissolution in 2 mL of phosphate-buffered saline (PBS, pH 7.4) under continuous agitation in a platform shaker for 2 h. After complete dissolution, the solution was filtered (0.22 μm nylon membrane) and diluted to a quantifiable concentration range. PAL-HCl content was analyzed using the validated UPLC method described in an earlier section, with triplicate (*n* = 3) measurements ensuring analytical precision.

### FTIR analysis

Fourier-transform infrared (FTIR) spectroscopy (IRAffinity-1 S, Shimadzu Corp, MD, USA) was conducted to assess molecular interactions between PAL-HCl and PVA grades. The formed MNs were crushed into a fine powder and analyzed alongside PVA polymers (particle-engineered 4–88, 5–88, and 8–88) and pure PAL-HCl. Spectra were acquired in transmission mode over a wavenumber range of 4000–400 cm⁻¹, averaging 100 scans per sample at 4 cm⁻¹ resolution. The Happ-Genzel apodization function was applied to optimize the balance between spectral resolution and noise suppression, as recommended for polymer-drug interaction studies. Characteristic functional group peaks (e.g., O–H stretching at 3200–3600 cm⁻¹, C = O stretching at 1650–1750 cm⁻¹) were compared across samples to confirm the absence of covalent interactions or structural alterations.

### Differential scanning calorimetry (DSC) analysis

Thermal analysis was performed using a Shimadzu DSC instrument (Shimadzu Corporation, Japan) to evaluate the compatibility of PAL HCl with the PVA matrix and assess drug-polymer stability post-VCM. The fabricated microneedles were ground into a fine powder, and approximately 2–5 mg of each sample was weighed into hermetically sealed aluminum pans. The samples were then subjected to a temperature ramp from 25 °C to 400 °C at a heating rate of 5 °C/min under a nitrogen purge flow of 20 mL/min. An empty aluminum pan, identical to the sample pan, served as the reference to ensure baseline stability. The thermograms were analyzed for phase transitions (e.g., melting) and thermal events indicative of drug-polymer interactions. Temperature calibration was validated using high-purity indium (99.99%, melting point: 156.6 °C, ΔHfus = 28.4 J/g), following established protocols for DSC instrument verification.

### Scanning electron microscopy (SEM) imaging

SEM was utilized to examine the morphology, dimensions, and spatial arrangement of individual MNs within the MN array. For sample preparation, the MN arrays were affixed onto SEM stubs using black, non-reflective double-sided adhesive tape to minimize background interference. The mounted samples were then placed into the SEM chamber for imaging. The acquired images were analyzed to determine key structural parameters, including needle height, base width, tip width, and the distance between adjacent microneedles.

### Histological evaluation

Histological analysis was conducted using hematoxylin and eosin (H&E) staining, following a previously established protocol. Skin samples, both untreated and those exposed to PAL-HCl-PVA MNs, were embedded in optimal cutting temperature (OCT) compound and frozen at − 80 °C for 20 min. Once frozen, the samples were mounted onto metal stubs with OCT compound and secured in position. Sectioning was performed using a Leica CM1860 cryostat, with blade angle, stage height, and slide positioning carefully adjusted to produce 10 μm-thick sections on Polysine™-coated slides (Globe Scientific, Inc., NJ, USA). The tissue sections were fixed with 1% (w/v) formalin for 15 min, stained using the H&E method, and preserved in xylene to prevent dehydration. Microscopic evaluation was carried out using a Leica DM 750 optical microscope equipped with a 20x objective lens to compare intact skin architecture with microchannels formed by the MN arrays.

### Confocal microscopy

Confocal laser scanning microscopy was employed to assess the penetration depth of the PAL HCl-loaded PVA MNs. Skin samples were treated with the MNs for 2 min, followed by topical application of Fluoresoft-0.35%^®^ fluorescent dye for another 2 min. Any excess dye was gently removed, and the samples were subsequently examined using a Leica SP8 confocal microscope equipped with a 10x air objective lens and an excitation wavelength of 496 nm. Z-stack imaging was performed with a 10 μm step size, beginning at the skin surface and continuing until the fluorescent signal was no longer visible. The insertion depth was calculated by multiplying the number of z-stack layers with the step size, providing a quantitative measure of microneedle penetration.

### Mechanical strength of PAL-HCl-PVA microneedles

The axial mechanical strength of microneedles was evaluated using a TA.XT Express Texture Analyzer (Stable Micro Systems, UK) to assess resistance to deformation under compressive forces. A single microneedle array was affixed to a 7 mm stainless steel probe with needles oriented downward. The probe descended at 0.1 mm/s in cycle test mode, applying trigger forces of 25 N (simulating thumb pressure during application) and 50 N (representing extreme stress conditions) to induce controlled deformation (*n* = 3).

Post-test, microneedle height reduction was measured using SEM. SEM imaging confirmed needle deformation patterns, correlating mechanical strength with insertion capability.

### In vitro release testing (IVRT) of PAL HCl-loaded MNs

A cellulose dialysis membrane (14 kDa molecular weight cutoff) was employed to evaluate the in vitro release kinetics of PAL HCl from PVA MN arrays. Membranes were cut into 1 × 1-inch pieces, pre-soaked in PBS, pH 7.4, overnight, and mounted on vertical Franz diffusion cells (PermeGear, Inc., PA, USA; 5 mL receptor volume, 0.64 cm² diffusion area). The receptor chamber was maintained at 37 °C using a circulating water bath, while the membrane surface temperature was stabilized at 32 °C ± 1 °C to mimic skin conditions.

MN arrays (*n* = 4 per PVA grade) were positioned in the donor compartment with needles oriented upward to prevent membrane damage. The donor chamber was filled with 500 µL of PBS as dissolution media and was sealed with Parafilm^®^ to minimize evaporation. Aliquots (300 µL) were collected at predetermined intervals over 72 h, replaced with fresh PBS, and analyzed for PAL HCl content using the validated UPLC method.

### In vitro permeation testing (IVPT)

#### Skin integrity measurement

Dermatomed human skin was obtained from a tissue bank, shipped on dry ice, and stored at -80 °C until the morning of the in vitro permeation testing (IVPT) study. Prior to experimentation, the skin was thawed at room temperature for 10 min with the stratum corneum facing upward, followed by sectioning into pieces to fit the vertical Franz diffusion cells. The skin was equilibrated on the Franz cells for 15 min to stabilize tissue hydration.

Barrier integrity was evaluated via trans-epidermal electrical resistance (TEER) using a digital multimeter (Agilent Technologies, Santa Clara, CA, USA) [20, 21]. Skin samples exhibiting a resistance ≥ 15 kΩ-a threshold validated for intact barrier function in human skin models were selected for IVPT. For the “poke and solution” experimental groups, TEER measurements were repeated post-microporation to confirm successful stratum corneum disruption, shown by a drop in the resistance of the skin piece.

### IVPT groups

Dermatomed human skin was mounted on vertical Franz diffusion cells to evaluate the in vitro transdermal permeation of PAL HCl over a 72-hour period. PBS (pH 7.4) was used as the receptor medium. The receptor chamber temperature was maintained at 37 °C using a circulating water bath to ensure the skin surface temperature remained at 32 °C ± 1 °C, closely mimicking physiological conditions.

The study included seven groups (*n* = 4 each): (i) a control group, in which untreated skin was treated with 1% (w/v) PAL HCl in PBS; (ii) poke-and-solution groups, where 1% (w/v) PAL HCl in PBS was applied to skin pre-treated with blank microneedles (one blank MN per tested drug-loaded MN formulation, containing only the polymer); and (iii) test groups, in which PAL HCl-loaded PVA microneedles were inserted into the skin by applying uniform thumb pressure for two minutes. The microneedles were then secured in place using 3 M surgical tape before mounting the samples on the Franz diffusion cells.

At predetermined time intervals during the 72-hour period, 300 µL samples were withdrawn from the receptor compartment and immediately replenished with fresh PBS. The collected samples were analyzed for PAL HCl concentration using the validated UPLC method. These values were used to calculate the cumulative drug permeation and total drug permeation across the skin over time.

### Lag time calculation

Lag time was determined by identifying the most linear portion of the plot representing the cumulative amount of PAL-HCl permeated per unit area of human skin versus time. A linear regression was applied to this segment, and the lag time was calculated by extrapolating the line to the x-axis, where the y-value was set to zero.

### Stability testing

To evaluate the chemical stability of PAL-HCl-loaded PVA MNs under varying environmental conditions, samples were stored for three months at two distinct conditions: 25 °C/0% relative humidity (RH) and 40 °C/75% RH. The 25 °C/0% RH condition was maintained using a desiccator containing silica gel beads to ensure anhydrous conditions. For the 40 °C/75% RH condition, MNs in sealed glass vials were placed inside a desiccator (sealed without any silica beads) alongside a saturated sodium chloride (NaCl) solution, a well-established method to maintain 75% RH at 40 °C, and stored in a temperature-controlled oven. At predefined intervals over the study period, MNs were retrieved (*n* = 3), and PAL-HCl content was quantified using the validated UPLC method.

### Statistical analysis

Data analysis was performed using GraphPad Prism software (version 8.0.1; GraphPad Software, San Diego, CA). Results are expressed as mean ± standard error of the mean, based on experiments conducted in quadruplicate (*n* = 4). Differences among three or more independent groups were assessed using ordinary one-way analysis of variance (ANOVA). Tukey’s post hoc test was applied to adjust for multiple comparisons within the group. A p-value of less than 0.05 was considered statistically significant.

## Results

### Quantitative analysis

**Fig. 2:**
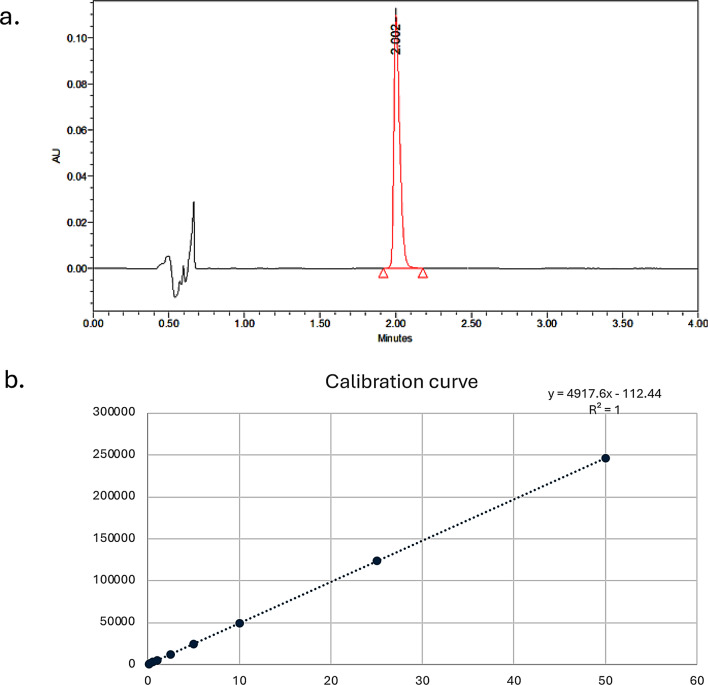
**a**) HPLC chromatogram for a 50 µg/mL concentration of PAL HCl in MeOH **b**) Calibration curve of PAL HCl

The UPLC method developed for the quantitative analysis of PAL HCl was validated for both interday and intraday precision and accuracy. Accuracy was within 100% ± 10%, and precision remained below 10% for both intraday and interday assessments. As shown in Fig. 2a, the UPLC chromatogram of a 50 µg/mL PAL HCl standard displays a distinct peak at approximately 1.9 min, detected at a wavelength of 240 nm. The method exhibited excellent linearity across the concentration range of 0.1 to 50 µg/mL, with an R² value of 1, as illustrated in Fig. 2b. The limit of detection (LOD) was determined to be 0.08 µg/mL.

### Slide crystallization

**Fig. 3:**
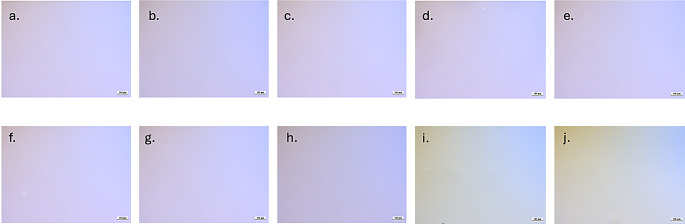
Optical microscopy images of the slide crystallization study. (**a**-**j**) PAL-HCL blends in PVA 4–88, Particle-engineered PVA 4–88, Particle-engineered PVA 3–82, PVA 5–88, PVA 8–88, PVA 18–88, PVA 26–88, PVA 40–88, Particle-engineered PVA 40–88, Particle-engineered PVA 5–88, respectively, showing no visible crystals

No crystals were observed in PAL-HCl-PVA blends containing 1–5% (w/w) PAL-HCl over a three-day period, as shown in Fig. 3.

### Fabrication of PAL HCl-PVA microneedles using VCM

Ten PVA grades- PVA 4–88, particle-engineered PVA 4–88, particle-engineered PVA 3–82, PVA 5–88, PVA 8–88, PVA 18–88, PVA 26–88, PVA 40–88, particle-engineered PVA 40–88, and particle-engineered PVA 5–88 (designated as M1–M10, respectively)- were used to make PAL HCl loaded film and further for microneedle fabrication feasibility using VCM (Table 1). Films containing more than 1% PAL HCl in various PVA grades failed to form microneedles, necessitating a standardized 1% PAL HCl loading across all formulations (M1–M10). Microneedle formation was strongly influenced by the viscosity and molecular weight (MW) of the PVA grades. PVA grades with very high viscosity (e.g., M6–M8) produced only partial needle formation or merely base structures, while very low viscosity grade M3 could not form MNs at all. Notably, as shown in the table, particle-engineered PVA 4–88 (M1) demonstrated superior performance compared to standard PVA 4–88 (M2), achieving deeper parafilm penetration and successful skin insertion capability. Formulations M4 (PVA 5–88) and M5 (PVA 8–88) also exhibited excellent microneedle formation with deep parafilm penetration and successful skin insertion.

**Table 1:**
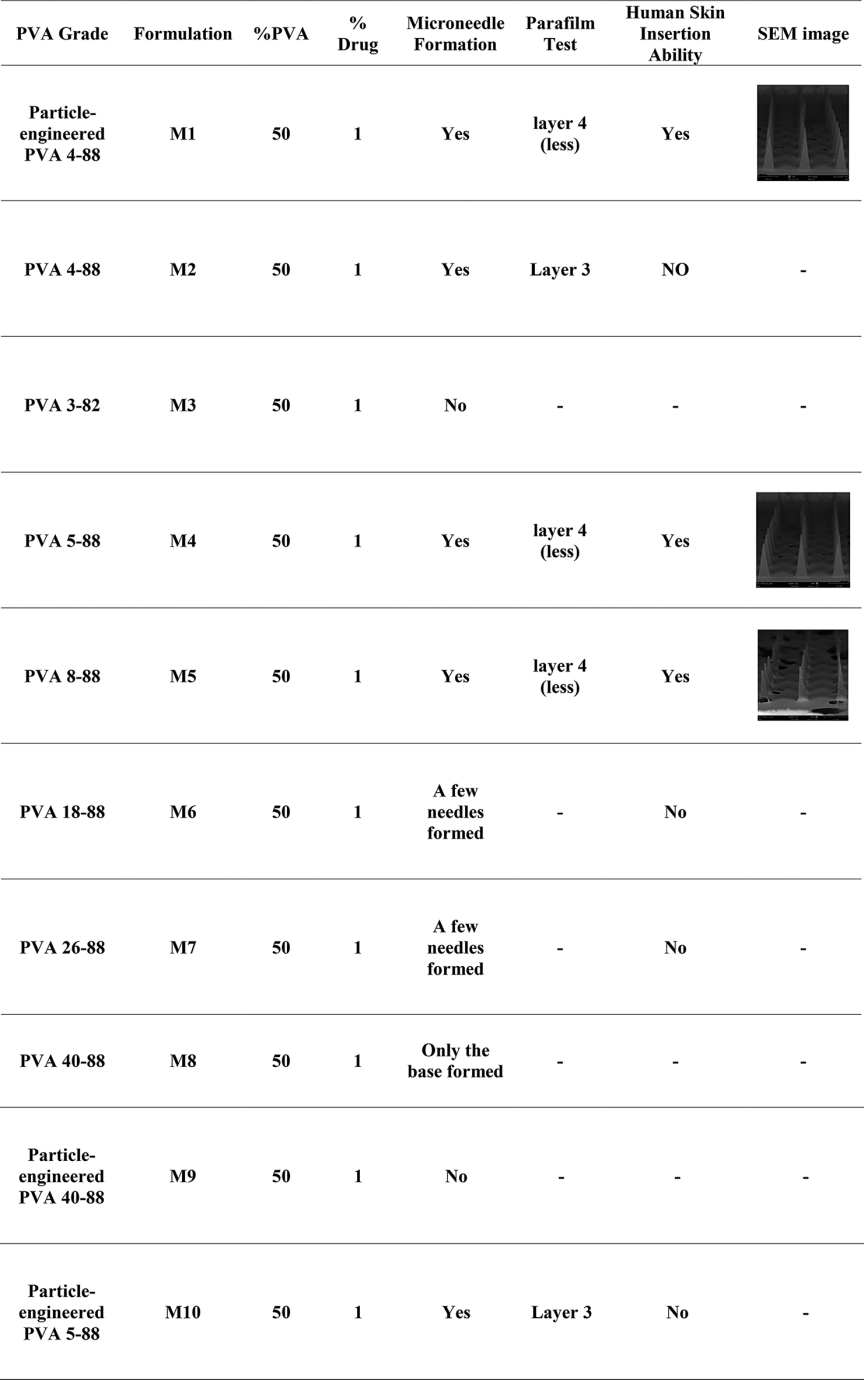
Formulation and characterization of the PAL HCl-loaded PVA microneedles

### Parafilm M^®^ insertion testing

**Fig. 4:**
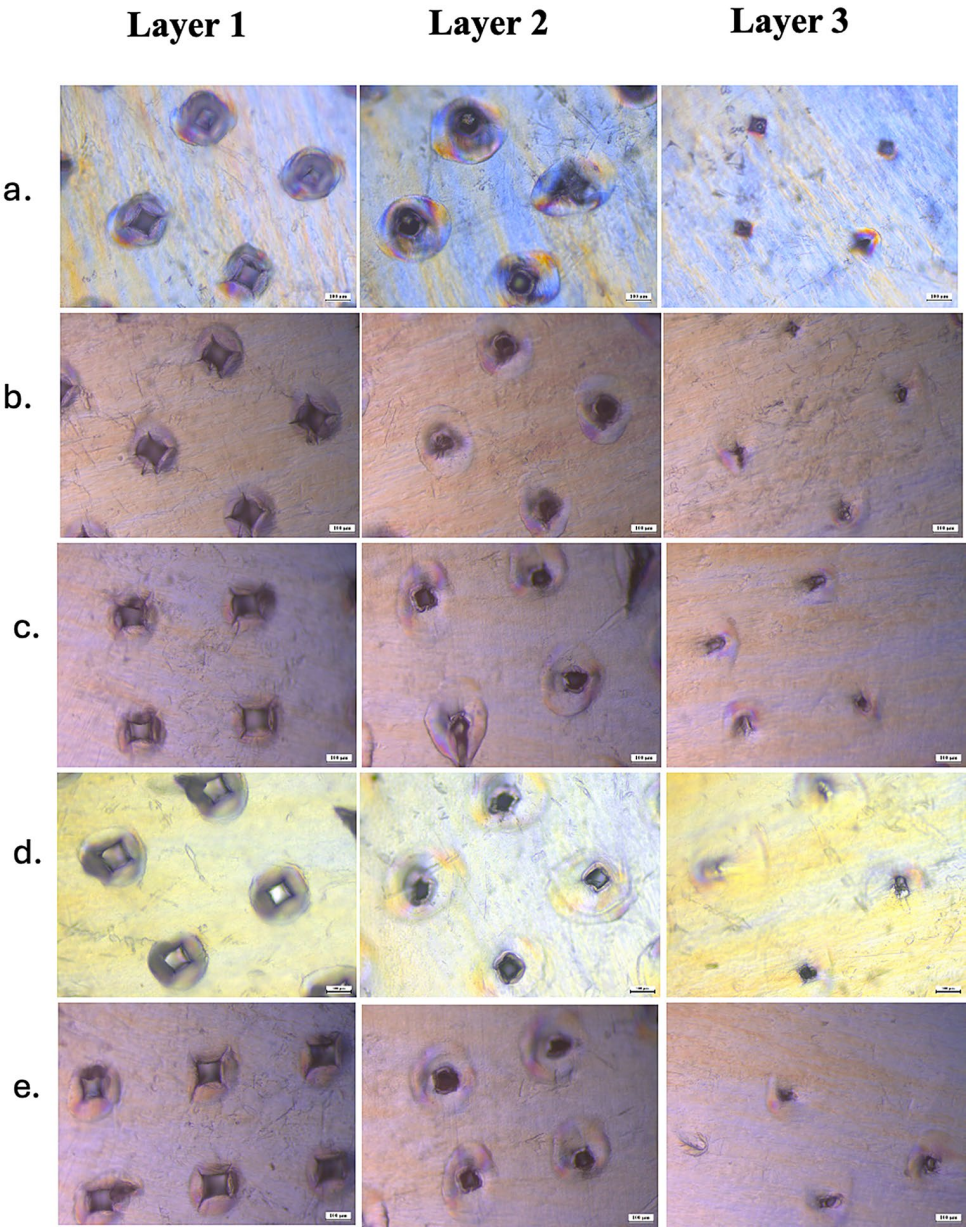
Parafilm^®^ insertion testing as a skin-simulant model for PAL-HCl-loaded PVA MNs **a**) M1, **b**) M2, **c**) M4, **d**) M5, and **e**) M10. Scale bar denotes 100 μm

Parafilm^®^ M, a skin simulant composed of hydrocarbon wax and polyolefin, was employed to assess microneedle penetration due to its elasticity and flexibility [22]. Microscopic evaluation of PAL-HCl-loaded PVA MNs (M1, M2, M4, M5, and M10) revealed consistent penetration through three Parafilm layers (Fig. 4), with a few of them achieving a fourth layer. The microchannels exhibited uniformity across MN arrays and all tested PVA grades, confirming reproducible structural integrity. A progressive reduction in microchannel diameter was observed through successive Parafilm layers, corresponding to the tapered geometry of the microneedles.

### Dye-binding studies for microchannel evaluation

**Fig. 5:**
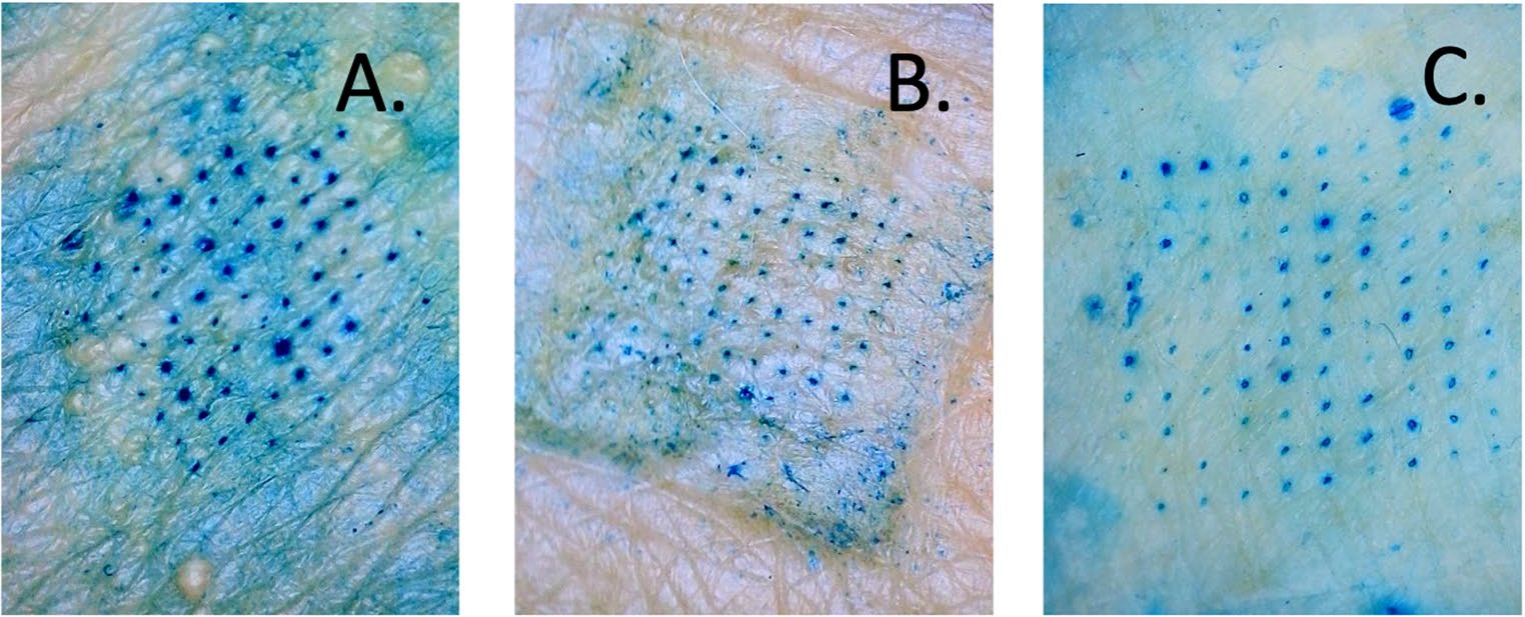
Methylene blue staining of (**A**) M1 (**B**) M4 (**C**) M5

Methylene blue staining was used to validate MN-mediated skin microporation and to assess penetration uniformity. The intact skin, protected by the hydrophobic stratum corneum, resisted methylene blue uptake due to the dye’s hydrophilic nature. In contrast, microneedle-treated skin showed distinct arrays of stained microchannels (Fig. 5), confirming successful disruption of the stratum corneum and the formation of hydrophilic pathways.

Based on the performance in Parafilm^®^ insertion testing and successful skin penetration, PAL HCl-loaded MNs M1 (particle-engineered PVA 4–88), M4 (PVA 5–88), and M5 (PVA 8–88) were selected for further evaluation.

### Drug content of the microneedles

The PAL HCl content in the selected MNs was quantified. M1 contained 1.78 ± 0.14 mg of PAL HCl, while M4 and M5 contained 1.78 ± 0.01 mg and 1.72 ± 0.09 mg, respectively.

### FTIR analysis

**Fig. 6:**
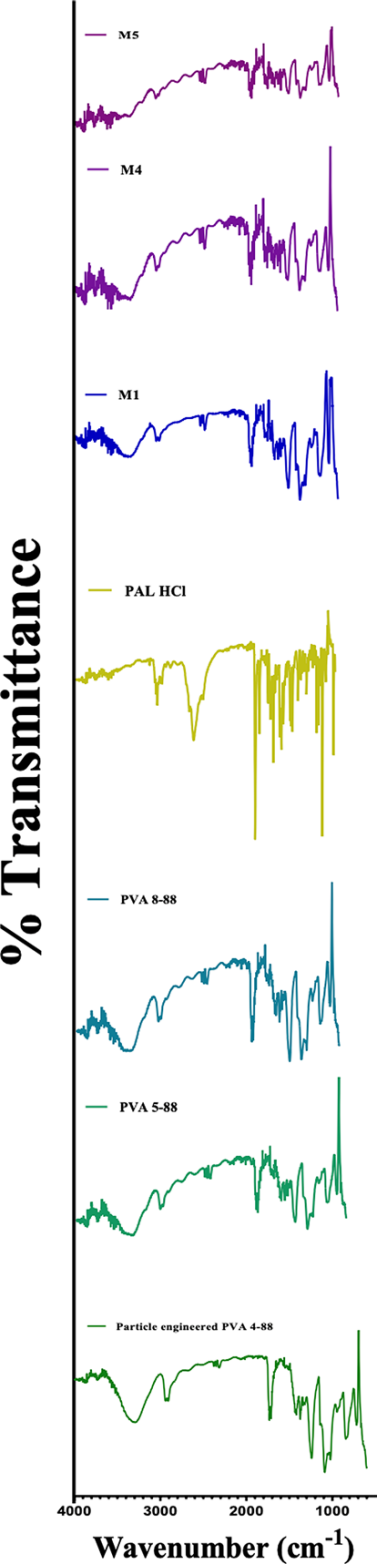
FTIR analysis of pure PAL HCl, PVA polymer grades (particle engineered 4–88, PVA 5–88, PVA 8–88), and PAL HCl-loaded microneedles (M1, M4, M5)

Figure 6 shows the FTIR spectra of pure PAL HCl, PVA polymers (particle-engineered PVA 4–88, PVA 5–88, and PVA 8–88), and the corresponding MN formulations (M1, M4, and M5). Pure PAL HCl exhibited characteristic absorption bands at 1650–1750 cm⁻¹ (C = O stretching), prominent sharp peaks in the 1450–1600 cm⁻¹ region (aromatic ring vibrations), and multiple characteristic peaks in the fingerprint region (1400 –500 cm⁻¹). All three PVA polymer grades (particle-engineered 4–88, 5–88, and 8–88) displayed similar spectral features, including a broad absorption band at 3200–3400 cm⁻¹ (O-H stretching), peaks at 2800–3000 cm⁻¹ (C-H stretching), and characteristic absorption patterns in the 1100 –1000 cm⁻¹ region (C-O stretching).

The microneedle formulations (M1, M4, and M5) exhibited spectra that contained all the characteristic absorption bands of both their respective PVA polymers and PAL HCl, without significant band shifts or the appearance of new peaks. The broad O-H stretching vibration from PVA was maintained in all MNs, while the distinctive PAL HCl peaks in the 1700 –1450 cm⁻¹ region remained visible. Minor intensity variations were observed across MNs, primarily due to overlapping of peaks seen due to transmittance at comparable wavenumbers. The preservation of all characteristic peaks confirms the absence of chemical interactions between PAL HCl and PVA polymers during the microneedle fabrication process, indicating good compatibility between the drug and polymer matrices.

### Differential scanning calorimetry (DSC)analysis

**Fig. 7:**
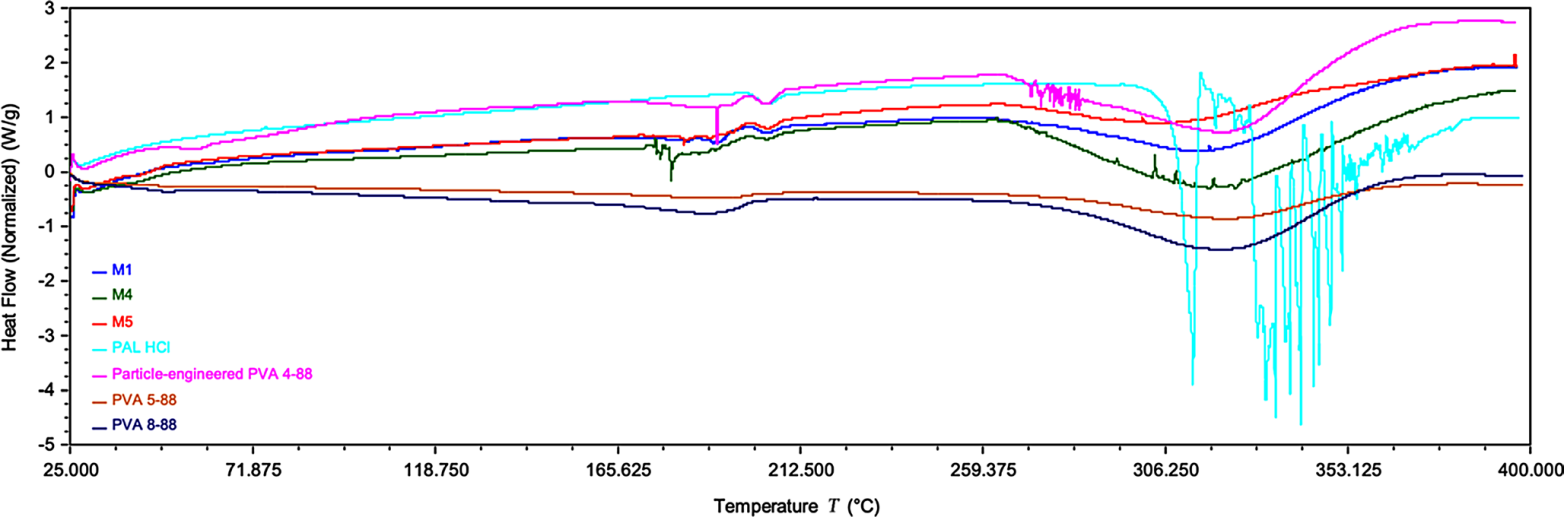
DSC thermograms of pure PAL HCl, PVA polymer grades (particle engineered 4–88, PVA 5–88, PVA 8–88), and PAL HCl-loaded microneedles (M1, M4, M5). Analysis performed from 25 °C to 400 °C at 5 °C/min heating rate under nitrogen atmosphere

DSC thermograms revealed critical insights into the thermal behavior and stability of PAL HCl-PVA MNs following exposure to high temperatures during VCM process (Fig. 7). Pure PAL HCl exhibited a sharp endothermic peak at 310–350 °C, with multiple sharp endothermic peaks indicating its crystalline melting behavior, consistent with its reported melting point of > 290 °C. In contrast, the particle-engineered PVA 4–88 and standard PVA grades (5–88, 8–88) displayed broad endothermic transitions between 300 and 330 °C. Notably, MNs (M1, M2, M4, M5) showed complete absence of the PAL HCl melting endotherm, potentially indicating amorphous dispersion or molecular-level interactions between the drug and polymer. The thermal profiles of the MNs closely mirrored their respective PVA components. The absence of distinct PAL HCl melting peaks in the MNs confirms successful integration of the PAL HCl into the PVA matrix to form MNs during vacuum compression molding, with no evidence of chemical degradation throughout the temperature range (25–400 °C).

### SEM imaging

**Fig. 8:**
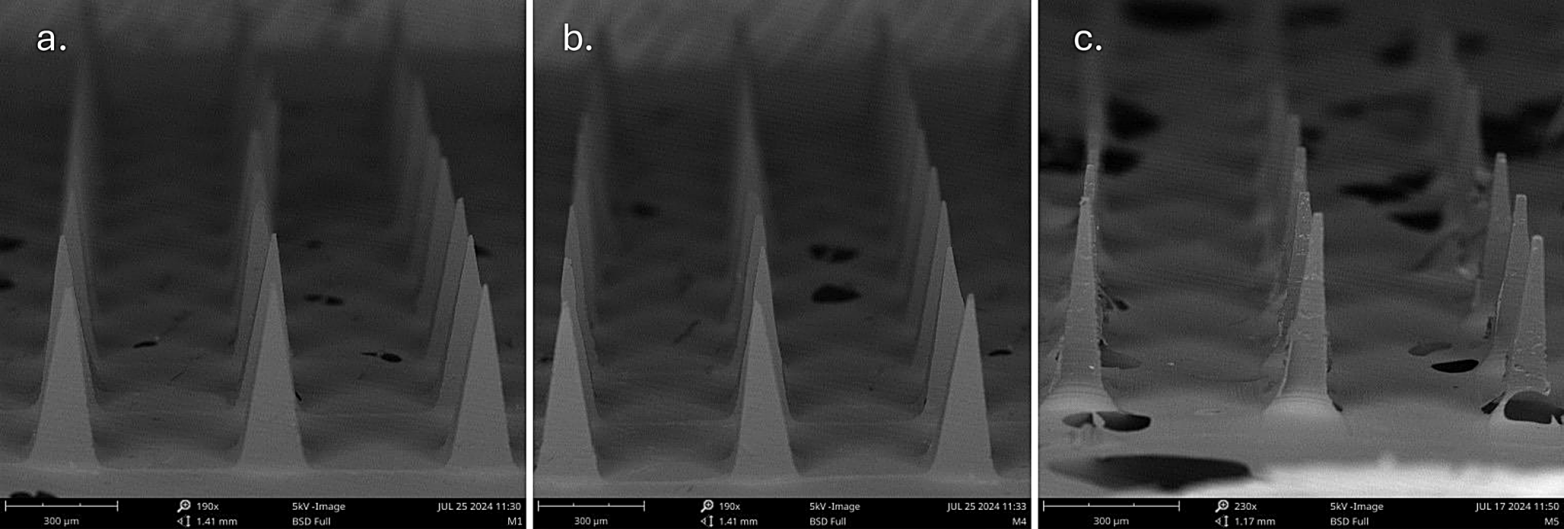
SEM images of **A**. M1 **B**. M4 **C**. M5

SEM was performed to assess the morphological and dimensional characteristics of the square pyramidal MN arrays. As shown in Fig. 8, the arrays exhibited structural uniformity across all fabricated units. Key geometric parameters- MN height, base width, tip width, and center-to-center interspacing distance- were measured in triplicate using SEM imaging. These measurements are summarized in Table 2.

**Table 2:** Dimensions and mechanical deformation characteristics of PAL HCl-loaded PVA microneedles (*n* = 3)

Microneedles	Needle height (𝝁m)	Needle base (𝝁m)	Interspacing distance (𝝁m)	Needle tip width (𝝁m)	% Height Reduction (25 *N* axial force)	% Height Reduction (50 *N* axial force)
M1	450.00	167.67	364.00	13.25	11.22	28.52
M4	449.33	186.00	369.33	13.73	5.79	15.06
M5	493.00	160.67	322.00	16.93	25.56	47.46

### Histological evaluation

**Fig. 9:**
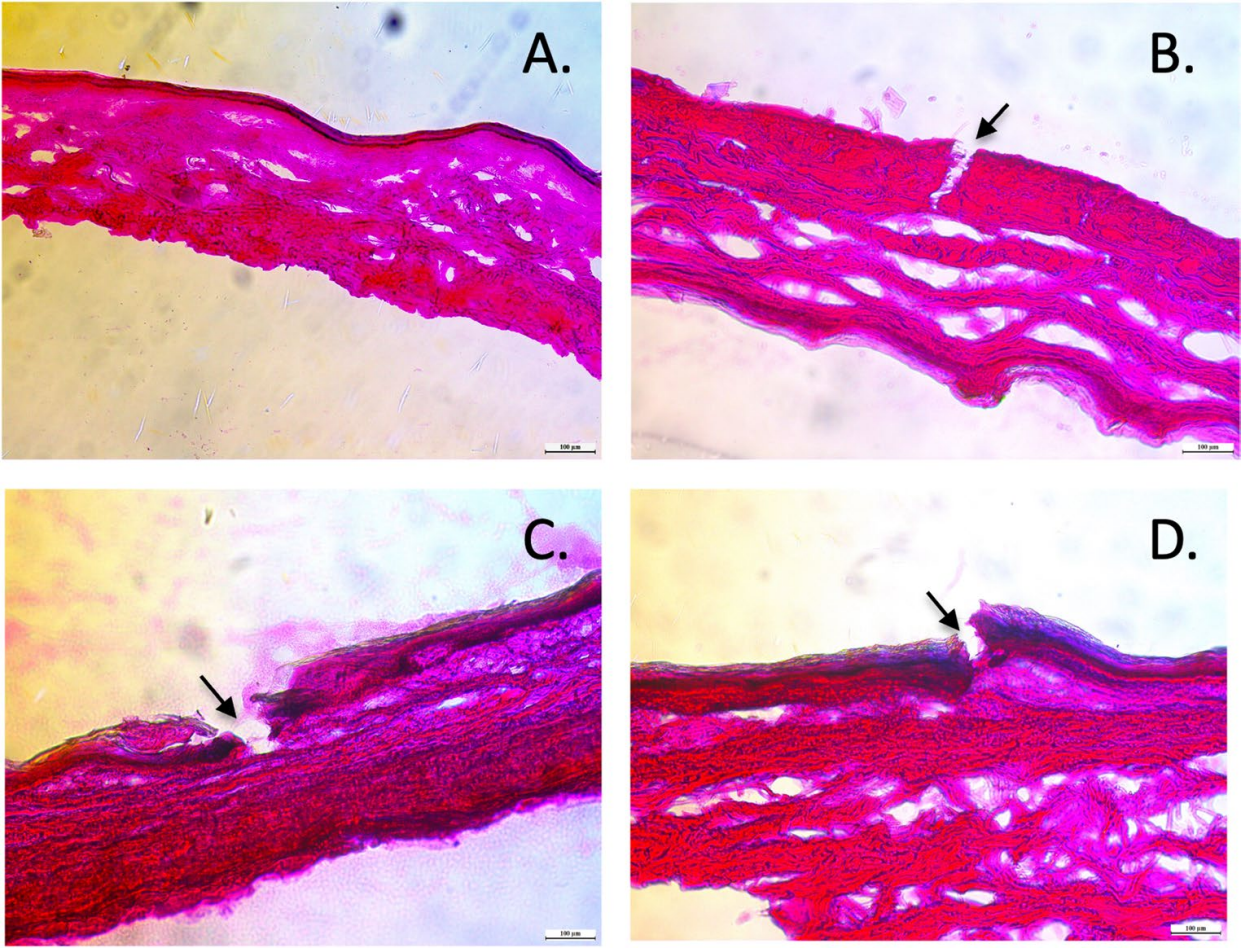
Histological evaluation of **A**. intact human skin and **B**. M1-treated human skin **C**. M4-treated human skin **D**. M5-treated human skin

Histological evaluation confirmed successful microporation of human skin following treatment with PAL HCl-loaded PVA MNs. Figure 9A shows the control (untreated) skin sample with intact stratum corneum, clearly defined epidermis, and underlying dermis layers with normal tissue architecture. The microchannels created by M1, M4, and M5 (corresponding to Fig. 9B and C, and 9D, respectively) demonstrated comparable penetration profiles. This consistency across the three different PVA grades suggests equivalent barrier disruption capabilities, indicating that molecular weight and viscosity variations between the polymers did not significantly impact their ability to create transdermal pathways for PAL HCl delivery.

### Confocal microscopy

**Fig. 10:**
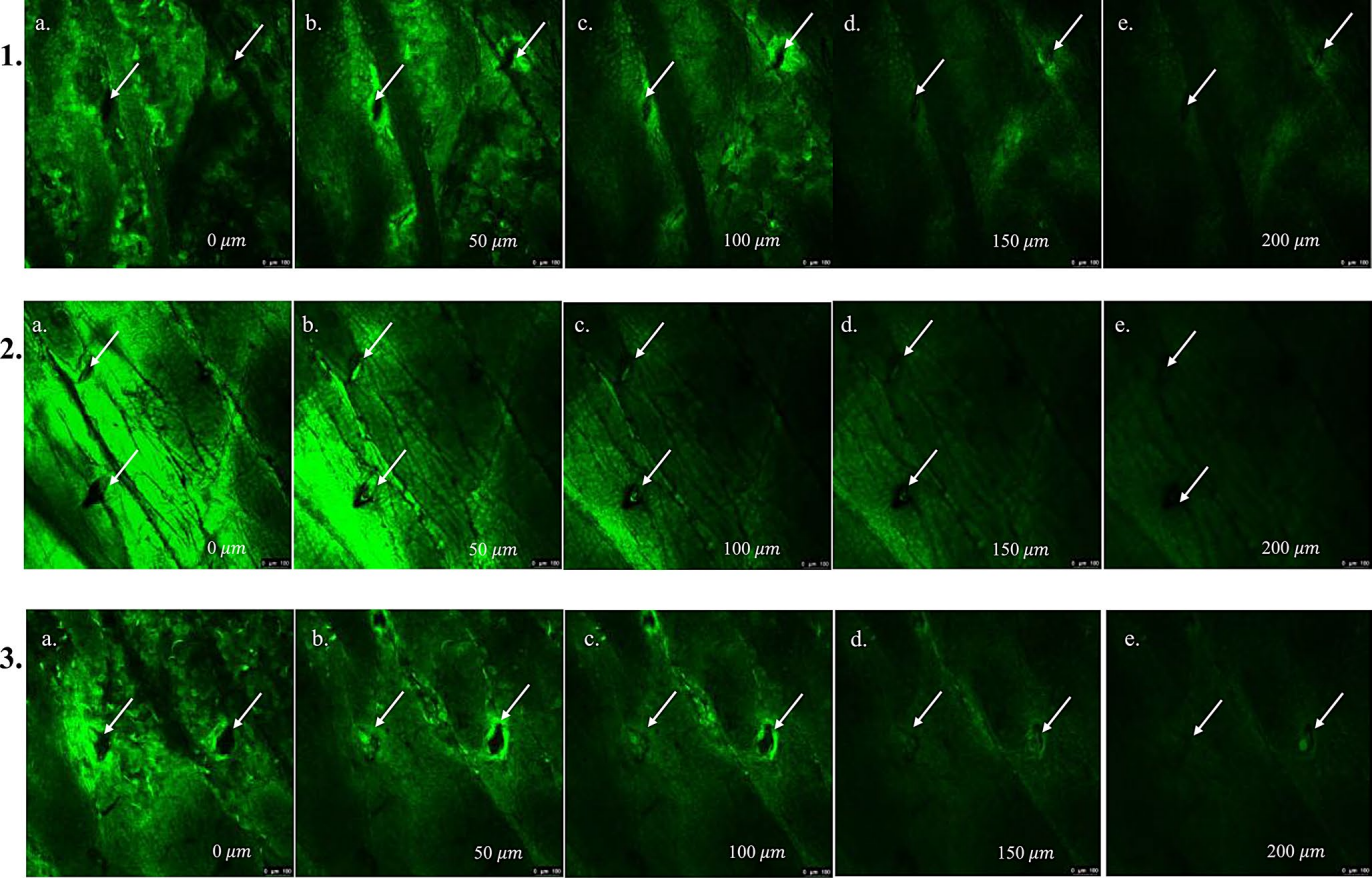
Confocal microscopy of human skin treated with PAL HCl-loaded PVA MNs **a**) M1, **b**) M4, **c**) M5

Confocal microscopy was employed to visualize and analyze the penetration depth of PAL HCl-loaded PVA MNs into dermatomed human skin (Fig. 10). As indicated by the white arrows, well-defined microchannels were observed across all MN groups (rows 1–3), with fluorescence signals detectable throughout the entire 200 μm imaging depth. Notably, all three PAL HCl-loaded PVA MNs exhibited a comparable penetration depth of 200 μm, with consistent fluorescence intensity observed at corresponding depths. This uniformity in penetration suggests that the different PVA grades used in this study offer similar performance in terms of skin penetration, despite their varying molecular weights and viscosities.

### Mechanical strength of PAL-HCl-PVA microneedles

The mechanical strength of PAL-HCl-PVA microneedles was assessed via axial compression cycle testing under forces: 25 N (simulating thumb pressure during application) and 50 N (representing extreme stress conditions). Height reduction served as the primary measure for deformation resistance. M1 and M4 exhibited superior mechanical integrity, with minimal height reductions of 11.22% and 5.79% at 25 N and 28.27% and 15.06% at 50 N, respectively, while M5 displayed susceptibility to compression (25.56% at 25 N; 47.46% at 50 N), shown in Table 2 (*n* = 3).

**Fig. 11:**
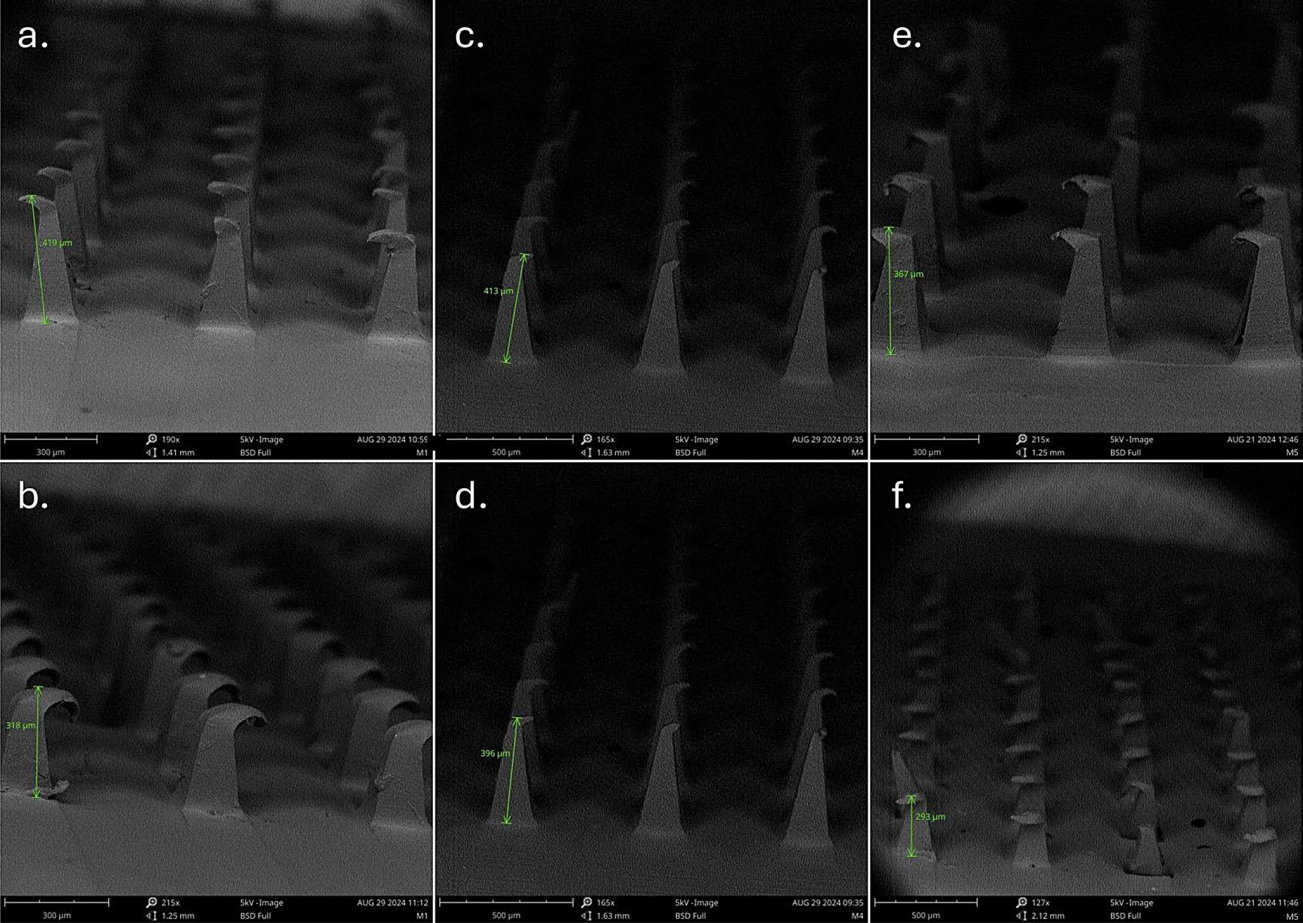
SEM images showing the mechanical deformation of microneedles after force application: (**a**, **b**) M1 subjected to 25 N and 50 N forces, respectively; (**c**, **d**) M4 subjected to 25 N and 50 N forces, respectively; (**e**, **f**) M5 subjected to 25 N and 50 N forces, respectively. Images demonstrate the decrease in microneedle height as a function of applied force

SEM imaging (Fig. 11a-f) corroborated these findings, revealing progressive deformation patterns across MN formulations. At 25 N (Fig. 11a, c, e), MNs retained structural integrity with minor tip compression, whereas 50 N (Fig. 11b, d, f) induced pronounced bending and buckling, particularly in M5.

These results highlight the critical role of polymer selection in MN design. M4 and M1, with height reductions below the 10–15% threshold established for polymeric MN systems, demonstrated optimal robustness and met the mechanical criteria for transdermal drug delivery, balancing structural integrity with functional performance.

### In vitro release testing (IVRT) of PAL HCl-loaded MNs

**Fig. 12:**
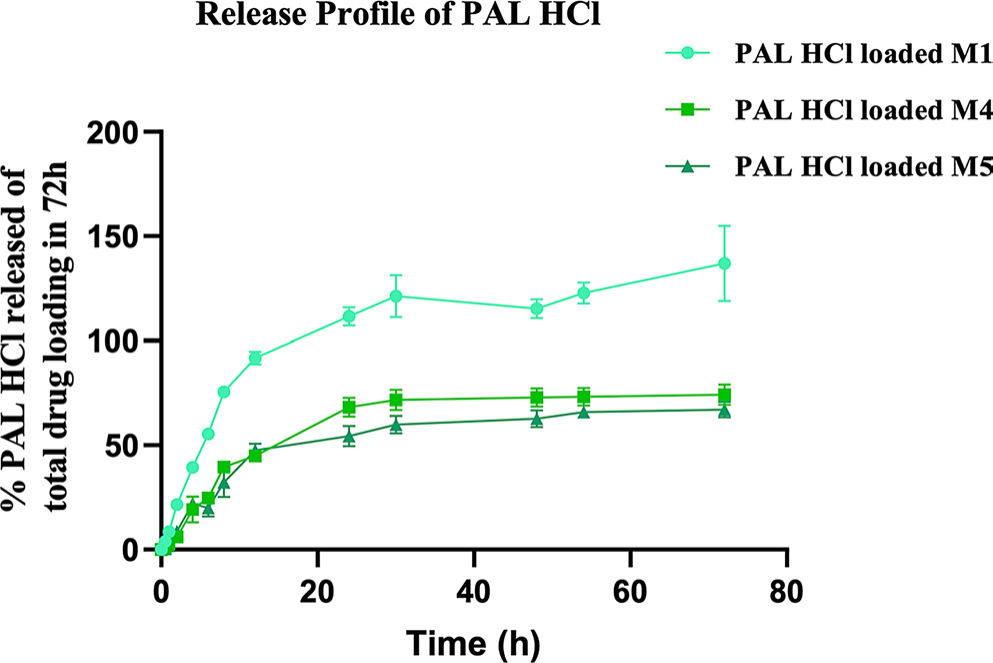
IVRT results: Release profiles for PAL HCL-loaded PVA MNs over a 3-day duration across cellulose dialysis membrane

IVRT studies were conducted to evaluate the release profile of PAL HCl from MNs made of various PVA grades over 72 h (Fig. 12). The results demonstrated that the percentage of drug released was influenced by the PVA grade used in the MNs. The particle-engineered PVA 4–88 formulation (M1) exhibited the highest release, with 100% of the total drug released within 72 h. In comparison, M4 (PVA 5–88) and M5 (PVA 8–88) showed lower percentages of drug release, reaching about 74% and 67%, respectively, over the same period. Notably, a substantial portion of the drug was released within the first 8 h for all MNs, followed by a more gradual and sustained release phase up to 72 h. These findings indicate that MNs fabricated with lower viscosity and molecular weights of PVA facilitated greater drug release, while higher viscosity and molecular weight grades resulted in slower, more controlled release profiles (M1 > M4 > M5).

### In vitro permeation testing (IVPT)

**Fig. 13:**
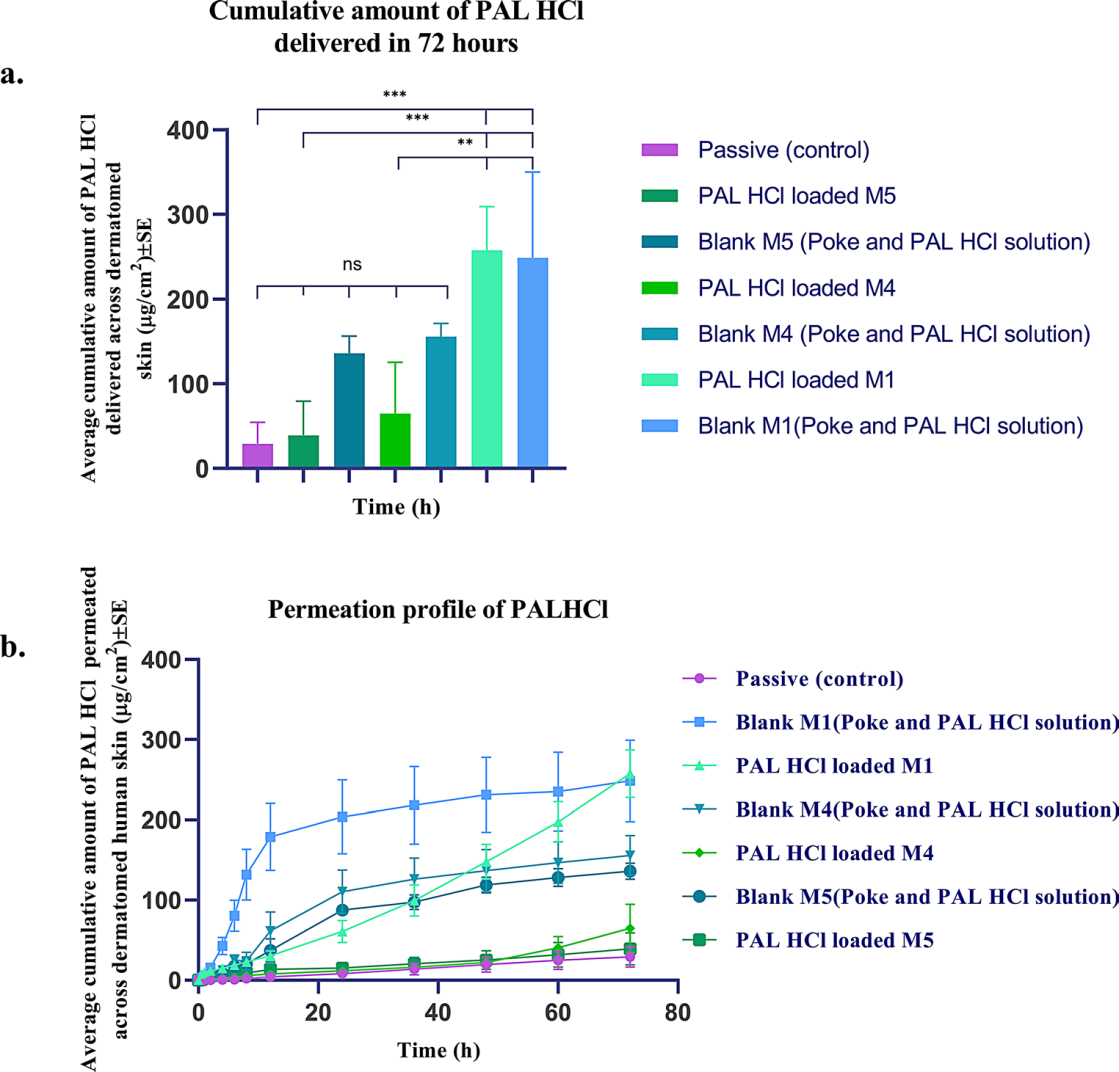
(**a**, **b**) Results from IVPT studies (*n* = 4); One-way ANOVA with Tukey’s post hoc analysis; ns (*p* > 0.05); **p ≤ 0.01; *** (*p* ≤ 0.001), Permeation profile of PAL HCl from MNs made of various PVA grades over 3 days

IVPT studies were conducted across dermatomed human skin to evaluate the transdermal delivery of PAL HCl from various PVA MNs (*n* = 4). The control groups (*n* = 4 each) evaluated included passive diffusion (1% PAL HCl solution in PBS applied to intact skin) and three “poke and solution” controls where skin was first micro punctured with blank MNs (M1, M4, or M5) followed by application of PAL HCl PBS solution. As illustrated in Fig. 13, the passive diffusion control group showed minimal permeation (29.10 ± 12.69 µg/cm²) over 72 h. The “poke and solution” groups demonstrated enhanced delivery: 248.42 ± 50.85 µg/cm² (blank M1), 155.93 ± 24.77 µg/cm² (blank M4), and 136.21 ± 10.14 µg/cm² (blank M5), with no significant differences among these blank MN treatments, confirming comparable microchannel formation across all three blank MN formulations facilitating similar PAL HCl delivery.

The PAL HCl-loaded PVA microneedles exhibited distinct sustained release profiles over 72 h, with permeation rates inversely proportional to the molecular weight and viscosity of the PVA grade used. M1 (particle-engineered PVA 4–88) achieved significantly higher cumulative delivery (257.56 ± 29.73 µg/cm²) compared to the passive diffusion group and as well as the higher molecular weight formulations M4 (64.99 ± 30.23 µg/cm²) and M5 (39.03 ± 20.20 µg/cm²). The permeation profiles shown in Fig. 13 clearly demonstrate this molecular weight and viscosity-dependent effect (M1 > M4 > M5), with M1 providing both more rapid initial permeation and higher cumulative delivery compared to M4 and M5.

### Lag time

Passive diffusion through intact skin exhibited the longest lag time (12.45 ± 2.16 h), reflecting the inherent barrier properties of the stratum corneum. Microneedle-mediated delivery substantially reduced this delay, with the blank M1, M4, and M5 poke-and-solution methods showing the shortest lag times of 1.76 ± 0.38, 1.21 ± 0.31, and 1.28 ± 0.25 h, respectively. PAL HCl-loaded M1, M4, and M5 MNs demonstrated a lag time of 2.89 ± 0.53, 3.84 ± 0.92, and 3.35 ± 0.29 h, suggesting that initial drug release kinetics are influenced by polymer dissolution and diffusion from the PVA matrix. The approximately seven-fold reduction in lag time for drug-loaded MNs compared to passive diffusion highlights their ability to bypass the stratum corneum barrier and accelerate therapeutic onset.

### Stability testing

Stability studies were conducted to assess the structural integrity and drug content retention of PAL HCl-loaded microneedle formulations (M1, M4, and M5) under two storage conditions: room temperature (RT) with 0% humidity and accelerated conditions (40 °C, 75% humidity) over three months. All three MNs maintained structural stability and consistent drug content during the first two months of storage. By the third month, formulation M1 retained both structural integrity and drug content under both storage conditions. In contrast, M5 exhibited minor structural changes but preserved its drug content. Formulation M4, however, showed a significant 98% reduction in PAL HCl content at the end of Month 3 under both storage conditions, with changes in both structure and solubility. These findings suggest that while M1 and M5 demonstrate robust stability, M4 may require formulation optimization to improve long-term drug retention.

## Discussion

Industry projections suggest the global market for MN-based TDS will reach approximately $10.9 billion by 2033 [23]. This remarkable growth trajectory reflects increasing recognition of MN technology as an extensively researched and well-funded drug delivery approach, evidenced by the significant number of ongoing clinical investigations [24]. Unlike conventional oral medications and injections, MNs offer several key benefits [25–27]. Particularly promising are drug-loaded polymeric MNs that can maintain therapeutic effects from 24 h to 4 weeks. These innovative MNs effectively overcome traditional treatment challenges by reducing dosing frequency, providing more consistent drug plasma levels, and supporting better treatment adherence through their controlled-release capabilities [22, 28].

In this study, VCM was employed for fabricating drug-loaded MNs, representing an improvement over previous MN-based TDS for PAL HCl and other hydrophilic therapeutics. Earlier methods primarily relied on solvent-dependent casting or micromolding, which involved lengthy drying, residual solvent risk, and multi-step processes that hinder scalability and reproducibility, especially for aqueous polymers like PVA [13]. To the best of our knowledge, this is the first work to report VCM technology to produce drug-loaded MNs.

In the VCM process, PDMS molds were used, which were fabricated by curing a silicone elastomer (e.g., Sylgard 184) at elevated temperatures (typically 60–150 °C) to achieve precise replication of microneedle geometries [29]. Unlike solvent casting, which requires prolonged drying (24 h–up to a few days) to remove organic residues, VCM eliminates solvent use entirely, reducing total fabrication time to under two hours per MN array. This is achieved through a streamlined two-step protocol: (1) heating polymer-drug blends under vacuum (-15 psi) to facilitate flow into mold cavities, followed by (2) rapid cooling [13]. The heating cycle was optimized to 260 °C for 15 min to ensure complete melting and flow of the PAL-HCl-PVA film into the mold cavities during MN fabrication using VCM, while preserving the structural integrity of PVA and the stability of PAL HCl. Initial attempts to directly add PAL HCl and PVA in the VCM chamber resulted in non-uniform PAL HCl-PVA mix and also drug degradation (charring) at high temperatures, needing a two-step process: (1) preparation of a homogeneous PAL HCl-PVA blend film, followed by (2) VCM processing of the film into MNs. This approach prevented thermal degradation of the drug while ensuring uniform distribution within the polymer matrix.

50% (w/w) PVA and 1% (w/w) PAL HCl were optimized as the optimal ratio for producing mechanically robust microneedles across most PVA grades. Maintaining a consistent 50% PVA concentration across different grades allowed for a comparison, ensuring that any variations in MNs’ performance, mechanical strength, and drug release were due to the intrinsic properties of each polymer type rather than differences in concentration. Higher MW and viscosity grades (e.g., PVA 18–88, 26–88, 40–88) exhibited reduced processability, likely due to insufficient polymer flow during molding.

Among the PVA grades evaluated, PVA 3–82 (82% hydrolysis) and nine 88% hydrolyzed variants (4–88, 5–88, 8–88, 18–88, 26–88, 40–88, and particle-engineered 4–88, 5–88, and 40–88) were investigated. Partially hydrolyzed grades (80–88%) are known to have superior water solubility, flexibility, and adhesion to hydrophobic surfaces compared to fully hydrolyzed PVA grades (>90%), attributed to their optimal hydroxyl-to-acetate ratio, which reduces crystallinity and enhances hydrogen bonding with water. Higher MW and viscosity grades exhibited reduced aqueous solubility due to increased polymer chain entanglement, though all PVA grades achieved complete dissolution at 50% (w/w) PVA while retaining 1% (w/w) PAL HCl [19, 30]. FTIR spectroscopy confirmed the absence of covalent interactions between PVA and PAL HCl, with preserved functional groups. DSC revealed PAL HCl remained amorphous within the PVA matrix, with no degradation peaks observed despite high-temperature VCM processing, confirming thermal stability.

Five MNs successfully retained structural integrity: M1 (particle-engineered 4–88), M2 (PVA 4–88), M4 (PVA 5–88), M5 (PVA 8–88), and M10 (particle-engineered 5–88). Higher MW/viscosity grades (e.g., 18–88, 26–88, 40–88) and lowest MW/viscosity grade PVA 3–82 failed to form intact MNs, likely due to insufficient polymer flow during molding. Post-penetration testing (Parafilm^®^ and dermatomed human skin), M1, M4, and M5 were used for further studies. M2 (PVA 4–88) and M10 (particle-engineered 5–88) exhibited inadequate penetration, suggesting particle engineering enhances processability for lower-MW grades (e.g., 4–88) but may complicate higher-MW formulations. Mechanical and structural analyses revealed no significant differences among M1, M4, and M5. SEM confirmed uniform needle geometry and structural integrity. Minor surface voids visible in the SEM images are attributed to air bubble entrapment within the PVA base during the vacuum compression molding process and do not reflect actual defects in microneedle formation. Complete insertion and penetration observed in human skin insertion tests confirm that these features had no impact on microneedle functionality or delivery performance. Texture analysis and height reduction tests demonstrated M1 and M4 having better mechanical strength, outperforming M5 marginally. Histological evaluation validated consistent stratum corneum disruption, while confocal microscopy confirmed penetration depths of ~ 200 μm. Hence, all three MNs were further explored using IVRT studies.

IVRT was conducted to understand the drug release kinetics of the PVA MN formulations using a dialysis membrane mounted on Franz diffusion cells. Unlike conventional methods, which often suspend MNs in a dialysis bag and add it to a large volume of continuously stirred media in a beaker, potentially leading to non-uniform drug distribution and unrepresentative sink conditions, this approach utilized a finite volume of release medium within the donor chamber, more closely simulating the limited interstitial fluid present in vivo (skin layers) [25, 31]. The MNs were added to the donor compartment, submerged in PBS solution with needles facing upward, and drug release was monitored as PAL HCl permeated across the membrane into the receptor chamber, where samples were collected and analyzed at predetermined intervals. Over three days, the release profiles revealed a rapid release phase for the first 8 h, followed by a sustained release up to 72 h. Importantly, the extent and rate of drug release were inversely related to the molecular weight and viscosity of the PVA used: M1 (particle-engineered PVA 4–88) achieved complete (100%) drug release within 72 h, while M4 (PVA 5–88) and M5 (PVA 8–88) released 74% and 67% of their drug loading, respectively. This trend is consistent with the lower solubility and slower dissolution kinetics of higher molecular weight and viscosity PVA matrices, which impede the release of the drug. These findings demonstrate that the release kinetics of PAL HCl from PVA MNs can be modulated by selecting appropriate polymer grades, enabling tailored delivery profiles for different therapeutic applications. These findings align with previous studies that reported grade-dependent processability and dissolution behavior in PVA MN systems [19].

IVPT studies revealed critical insights into PAL HCl delivery across dermatomed human skin. The control group (PAL HCl solution applied to intact skin) exhibited negligible permeation, consistent with the drug’s hydrophilic nature and the stratum corneum’s barrier properties. For poke-and-solution groups, pretreatment with blank microneedles (M1, M4, M5) followed by topical drug application showed no statistically significant differences among them in permeation, confirming consistent microchannel formation across PVA grades. In contrast, PAL HCl-loaded microneedles demonstrated MW and viscosity-dependent permeation profiles, with lower-MW/viscosity formulations (M1: particle-engineered PVA 4–88) achieving significantly higher cumulative permeation than higher-MW/viscosity MNs (M4: PVA 5–88; M5: PVA 8–88). Sustained permeation over 72 h correlated with gradual microneedle dissolution in the skin, where the released drug formed depots in the epidermal and upper dermal layers. The observed lag time (e.g., 2.89 ± 0.53 h for M1) aligns with prior reports of polymer-dependent diffusion kinetics, though further investigation is needed to separate formulation effects from skin barrier dynamics.

Compared to prior tip-loaded or coated MN strategies for PAL HCL, which emphasize immediate bolus delivery, VCM-fabricated MNs described in this study support modifiable burst-plus-sustained drug release profiles by precise selection of PVA molecular weight and grade. This enables both acute and extended therapeutic coverage for conditions like CINV, addressing limitations of earlier approaches that offered either rapid but short-term relief (MN bolus/iontophoresis) or slow, patch-like delivery (drug-in-adhesive TDS) [12, 14]. Notably, in this study, we used the VCM approach to fabricate drug-loaded MNs of PVA, yielding mechanically strong, with high skin penetration efficiency and tunable release characteristics over days, while previous blank PLGA MNs fabricated using VCM did not address drug loading, API stability, or grade-dependent functionality in hydrophilic matrices [13]. When compared to hydrogel-based and PLGA MNs, hydrogel MNs offer tunable, sustained drug release via swelling and water uptake but often have lower mechanical strength and increased risk of breakage. In contrast, PLGA MNs demonstrate robust mechanical properties and long-term drug release but require solvent-intensive, multi-step fabrication and are governed by slow polymer degradation [11, 13].

Stability studies showed variations among PVA microneedle formulations (M1, M4, M5), probably due to differences in MW, viscosity, and structural properties. M1 maintained structural integrity and drug content over three months, probably due to its lower MW and reduced crystallinity. In contrast, M4 (PVA 5–88) and M5 (PVA 8–88) maintained stability up to 2 months, exhibiting intermediate stability.

These findings underscore the potential of VCM for fabricating tunable, long-acting drug-loaded MN TDS. Future work should explore VCM’s applicability to other hydrophilic APIs and evaluate in vivo efficacy in preclinical models. Scaling production while maintaining batch consistency will be critical for clinical translation, particularly for chronic conditions requiring sustained drug delivery.

## Conclusion

This study demonstrated the feasibility of fabricating PAL HCl-loaded PVA MNs using a novel VCM technique. The findings reveal that intermediate molecular weight and viscosity grades of PVA are optimal for producing mechanically robust microneedles capable of reliable skin penetration and sustained transdermal delivery. Characterization by SEM, texture analysis, confocal microscopy, and histology confirmed that M1, M4, and M5 shared similar morphology, mechanical strength, and penetration profiles.

Sustained in vitro release of PAL HCl over 72 h was achieved, with drug release rates inversely related to polymer molecular weight and viscosity. The observed biphasic release pattern-characterized by an initial burst followed by gradual diffusion-supports the clinical need for both rapid onset and prolonged antiemetic action. Further, IVPT revealed that M1 provided significantly greater cumulative drug delivery (257.56 ± 29.73 µg/cm²) compared to both passive diffusion and higher molecular weight formulations (M4: 64.99 ± 30.23 µg/cm²; M5: 39.03 ± 20.20 µg/cm²). These results confirm that lower MW/viscosity, particle-engineered PVA enables enhanced permeation and rapid transdermal drug delivery.

Overall, this work highlights VCM as a robust and versatile platform for producing drug-loaded MNs with tunable release properties outperforming hydrogel-based MNs in stability and matching polymers like PLGA’s strength in MN fabrication, while offering simpler, solvent-free production.

The primary limitation of this study is the absence of in vivo data and dose optimization, which restricts the ability to fully assess the pharmacokinetics, safety, and therapeutic efficacy of the developed microneedle system. Future studies should focus on translating these findings to clinical settings, including pharmacokinetic evaluation and assessment of the method’s suitability for delivering biologics and thermally sensitive compounds.

## Data Availability

The datasets generated during and/or analyzed during the current study are included in the manuscript.

## Abbreviations

PVA: Polyvinyl alcohol
MNs: Microneedles
PAL HCl: Palonosetron hydrochloride
TDS: Transdermal delivery systems
SEM: Scanning electron microscopy
LOD: Limit of detection
LOQ: Limit of quantitation
IVRT: In vitro release testing
IVPT: In vitro permeation testing
PBS: Phosphate-buffered saline
VCM: Vacuum compression molding
TEER: Trans-epidermal electrical resistance
